# Socioeconomic, Behavioural, and Protective Factors Influences on the Combined Prevention of HIV Infection Among Brazilian Amazon Men Who Have Sex with Men: A Cross-Sectional Study

**DOI:** 10.3390/tropicalmed10080231

**Published:** 2025-08-16

**Authors:** Thiago Vilhena Silva, Iaron Leal Seabra, Glenda Roberta Oliveira Naiff Ferreira, João Gabriel Alves da Luz, Cecília Conceição Viana, Lucas Barros de Paiva, Glauber Weder dos Santos Silva, Caio Lacerda dos Santos, Luiz Fernando Almeida Machado, Eliã Pinheiro Botelho

**Affiliations:** 1 Programa de Pós-Graduação em Enfermagem, Universidade Federal do Pará, Belém 66075-110, Brazil; thiago.enf123@gmail.com (T.V.S.); iaronlealseabra@gmail.com (I.L.S.); glendaf@ufpa.br (G.R.O.N.F.); gabriel.alves.luz.31@gmail.com (J.G.A.d.L.); cecilia.viana@ics.ufpa.br (C.C.V.); luccasdbp@gmail.com (L.B.d.P.); glauberweder@hotmail.com (G.W.d.S.S.); caiolacerdasantos@hotmail.com (C.L.d.S.); lfam@ufpa.br (L.F.A.M.); 2 Faculdade de Medicina, Centro Universitário Metropolitano da Amazônia, Belém 66053-000, Brazil

**Keywords:** HIV, men who have sex with men, protective factors, combined prevention strategy, Brazil, Amazon

## Abstract

We analysed the socioeconomic, behavioural, and protection factors (PFs) influences on the HIV combined prevention (CP) strategy among Brazilian Amazonian men who have sex with men (MSMs). PFs are resources that reduce the effect of adversity and help people maintain their well-being. Methods: Cross-sectional study employing a convenient sample of MSMs living in the metropolitan region of Belém. A questionnaire containing socioeconomic, behavioural, PFs, and behaviour/knowledge concerning CP questions was used. “Behaviour/knowledge concerning CP” was defined as a dependent variable and received a maximum score of 16 points. The Mann–Whitney and Kruskal–Wallis tests and multiple linear regression were employed. Results: Our sample comprised 384 MSMs scoring an average of 7.83 points (±1.9). Contributing to lower scores were “not talking about sex life with confidants”, “not talking with work colleagues about personal life and sexually transmissible infections”, and “not participating in non-governmental organisations.” On the other hand, “not being happy in the neighbourhood of residency” contributed to higher scores. Conclusion: Peer support and social inclusion are essential for increasing MSMs’ access to CP.

## 1. Introduction

The population of men who have sex with men (MSMs) has been significantly affected by the Human Immunodeficiency Virus (HIV) epidemic. While HIV has a global prevalence of 0.8%, its prevalence among MSMs is as high as 7.7% [1]. In Latin American countries, the prevalence ranges from 1.2% to 32.6% [2]. In Brazil, considering all men aged ≥13 years, MSMs represent >50% of the new HIV notifications [3].

Since 2013, the combined prevention (CP) strategy has been recognised as the most effective approach for preventing HIV and other sexually transmitted infections. Combined prevention takes into account individuals’ needs and specificities, considering both their social context and stage of life. It addresses behavioural, biomedical, legal and structural aspects [4]. Biomedical strategies include technologies employed to eliminate HIV transmission, such as pre- and post-exposure prophylaxis (PrEP and PEP, respectively), condoms, antiretroviral therapy (ART), rapid testing for HIV and other STIs, vaccinations against hepatitis A and B (HAV and HBV, respectively), and vaccination against human papillomavirus (HPV). Behavioural strategies focus on reducing HIV-risk behaviours, including decreasing the number of sexual partners, reducing substance use, increasing condom usage and adherence to PEP, and promoting biannual HIV, syphilis, HBV, and hepatitis C testing. On the other hand, the legal and structural components focus on fighting stigma and discrimination that act as barriers to HIV prevention. For example, in Brazil, there is the channel “Dial 181” that allows individuals to complain to the public security offices in case of crimes, such as discrimination [5,6].

To facilitate access to CP, in addition to offering preventive and diagnostic HIV services in Testing and Counselling Centres (TCCs), the Brazilian Ministry of Health implemented several strategies and measures, such as the decentralisation of rapid tests, distribution of condoms, lubricant gel distribution, PrEP, and ARTs to the primary healthcare network (PHC) [7,8]. Furthermore, PEP is freely available in public healthcare facilities, such as hospitals, TCCs, and specialised HIV services [9].

Although all implemented strategies toward HIV prevention, and even with the Unified Health Brazilian System (SUS) and the National LGBT Policy establishing equal right to health for all, are free from prejudice and discrimination, as well as qualified professionals [10,11], men who have sex with men still face several barriers when accessing PHCs, such as discrimination, unqualified healthcare professionals to attend their specific needs, and low socioeconomic conditions [12,13].

Protective factors (PFs), defined as individual and environmental resources that reduce the impact of risks and adversities and help people preserve their well-being, can help overcome these barriers. These can be categorised into individual, interpersonal, institutional, community, and policy levels [14]. For example, a study among Black American MSMs and transgender women showed that having a parental confidant was associated with a greater chance of PrEP use, and among those living with HIV, the support of a confidant was directly associated with HIV viral suppression [15].

Studying the influence of socioeconomic, behavioural, and PFs in the CP strategy is essential to develop mechanisms to strengthen the implementation of CP among MSMs, reducing the detection rate of HIV and contributing to reaching the HIV elimination goal by 2030, as proposed by UNAIDS [16]. From 2000 to 2023, Brazil recorded 954,069 HIV cases, representing a prevalence of 0.4%. Studies addressing the association between PFs and CP are scarce in the Brazilian Amazon region, where the HIV detection rate is the highest in Brazil. MSM accounts for 45% of all HIV notifications, and the epidemic has been increasing since the 1980s [3]. Therefore, in this study, our main goal was to analyse the association between PFs and behaviour and knowledge toward CP among MSMs in the Brazilian Amazon.

## 2. Materials and Methods

### 2.1. Study Design and Settings

This was a cross-sectional study of MSMs living in the Belém metropolitan region (BMR). The Belém Metropolitan Region is located in the state of Pará, the second-largest state in Brazil, and consists of eight municipalities: Ananindeua, Barcarena, Belém, Benevides, Castanhal, Marituba, Santa Bárbara do Pará, and Santa Izabel do Pará. Its population is 2.5 million, approximately 38.8% of Pará’s population [17]. Of the population of the BMR, 31% live in conditions of extreme vulnerability [18].

Among the 100 Brazilian municipalities with the highest HIV detection rates, 4 were in the BMR: Belém (7th municipality), Castanhal (11th municipality), Marituba (10th municipality), Ananindeua (26th municipality), and Barcarena (89th municipality). In terms of monitoring people living with HIV and PrEP distribution, Belém has two specialised healthcare centres, one testing and counselling centre, and only two primary healthcare centres. All the other cities had only one healthcare facility that offered these services.

In this study, we used a formulary containing socioeconomic, behavioural, PFs, and CP questions (see Appendix A for Table A1 and Table A2). Protective factors questions were constructed based on a previous study [15]. Before collecting the data, we pretested the formulary with undergraduate students, and all data collectors were trained.

For the sample calculation conducted in EpiInfo versão 7.2.5.0 (CDC, Atlanta, GA, USA), we adopted an error of 5%, a confidence level of 95%, and an expected frequency based on a study of MSMs in Brazil showing a prevalence of inconsistent condom usage with steady partners of 64.5% with steady partner [19]. Owing to the lack of specific data on the MSM population in the IBGE Census and DATASUS for the BMR, the calculation was based on the male population of the BMR aged 15–59 years, at 406,720 inhabitants [20]. The calculation resulted in a sample size of 352 MSMs.

Because of the difficulty in reaching the goal population, we employed Time Location Sampling (TLS) methods and collected data from LGBT bars and parties. The questionnaire was completed in a more reserved and less noisy place, guaranteeing voluntary confidentiality and good communication with the interviewer. All volunteers signed consent forms to participate in the study.

### 2.2. Inclusion and Exclusion Criteria

The participants eligible for this research were MSMs, cisgender, residents of the BMR, aged ≥18 years, and self-declared as not having HIV.

Participants who were under the influence of psychoactive drugs at the time of data collection were excluded from the study.

### 2.3. Variables

The outcome variable “behaviour/knowledge” was made up of 15 questions related to CP (Table A1). For each item answered correctly, the volunteer received one point, except for question 15, which could score two points in the case of the volunteer answering that they were co-vaccinated against HBV and HPV. The maximum possible score was 16 points.

The independent variables comprised socioeconomic, behavioural, and PFs questions (Table A2).

### 2.4. Data Analysis

The data collected were entered into spreadsheets using Microsoft Office Excel^®^ 2019 software (Redmond, WA, USA), double-checked, and redundancies removed.

Descriptive statistics were used (Microsoft Office Excel^®^ 2019) and the results were presented as absolute (n) and relative (%) frequencies, averages with standard deviations (±), and medians.

To test the association of PFs with behaviour and knowledge concerning CP, first, we analysed separately the statistical difference in the median score among the factors comprising each PF employing Mann–Whitney and Kruskal–Wallis tests for two and three independent samples, respectively, to filter the variables to be analysed in multiple linear regression. The choice of these statistical tests was due to the fact that the dependent variable was codified in a score measured on an ordinal level. The protective factors that individually showed a *p*-value of <0.10 were then analysed by multiple linear regression analysis using the stepwise method. Only variables with *p* < 0.05 and a variance inflation factor (VIF) were considered. Additionally, we considered the regression coefficients (Coeff), 95% confidence intervals (CIs), R2, and adjusted R2 of the model. All analyses were performed using Minitab version 21 (LLC, State College, PA, USA).

## 3. Results

The study sample comprised 384 participants, with none of them dropping out after accepting the invitation and signing the free consent forms. The average score achieved by the participants with regard to the dependent variable was 7.83 points (±1.9), with minimum and maximum scores of 3 and 13 points, respectively, and a median of 8 points. Table 1 shows the absolute and relative frequencies of the volunteers who received each factor comprising the dependent variable. The majority of participants answered correctly about HIV and syphilis transmission routes (98.44% and 84.38%, respectively) and were aware of PrEP (59.11%), PEP (58.33%), and PHCs offering the rapid test (82%). However, a minority of the participants affirmed the constant use of condoms (21.4%), not using psychoactive substances (22.9%), being aware of HIV autotests (47.25%), TCCs (42.39%), and condom and lubricant distribution by PHCs (9.89%). Additionally, only 37.76% and 11.72% underwent at least one rapid test for HIV and syphilis, respectively, every 6 months, 0% were co-vaccinated against HPV and HBV, and 21.16% knew the “Dial 181” platform.

Table 2 shows the factors comprising the individual levels of PFs and the median scores. The majority of participants were >30 years old (75.52%), lived in Belém (63.28%), were White/Asian (50.52%), single (55.91%), had a family income of between two and four minimum wages (63.55%), had health insurance (57.82%), had no tattoos (68.72%) or piercings (70.34%), had not been sexually abused (96.36%), receive money for sex (52.34%), did not paid for sex (80.29%), did not have sex with people living with HIV (81.77%), and used dating applications (87.76%).

Concerning interpersonal level PFs, most of the volunteers were not religious (45.05%), rarely/sometimes/almost always talked with a confidant about their sexual life (57.56%), frequented socialised in places where they discussed STIs and HIV prevention (54.92%), knew and talked with neighbours (53.35%), hung out with friends or colleagues from the same neighbourhood (52.01%), were unemployed (59.63%), and were used to talking about their private lives and STIs with work colleagues (62.76%) (Table 3).

At the community level (Table 4), most participants reported living in a neighbourhood with leisure opportunities (53.16%), places to play sports (55.48%), adequate garbage collection (55.26%), basic sanitation (55.23%), and public transport (55.48%). Furthermore, the majority of them did not participate in non-government organisations (NGOs) (84.37%), participate in neighbourhood associations (50.26%), were happy in the neighbourhood (51.04%), and resided in the same house for more than six months (87.76%).

At the policy level, a larger number of volunteers affirmed voting for politicians supporting LGBT causes (54.96%), rarely/sometimes/almost always demanding actions from the politicians they elected (59.12%), knowing the national LGBT policy (55%), and the principles of the SUS (54.16%) (Table 5).

The variables with *p* < 0.10 were then analysed in the multiple linear regression analysis that indicated the contributors with lower scores to be “never talked with confidants about sex life” (Coeff = −0.586, *p* = 0.042), “not participating in NGOs” (Coeff: −0.758, *p* = 0.006), and “not talking about personal life and STIs with work colleagues” (Coeff = −0.459, *p* = 0.02). Conversely, not being happy in their neighbourhood contributed to higher scores (Coeff = 0.381, *p* = 0.048) (Table 6).

## 4. Discussion

The results of this study revealed that, although most participants said they were aware of the ways in which HIV is transmitted, they had low adherence to fundamental preventive practices. The majority of participants reported inconsistent condom use, lack of biannual HIV testing, substance use, as well as lack of knowledge about self-testing, TCCs, availability of condoms and lubricant gel at PHCs, and the “Dial 181” platform.

The sample was mostly made up of individuals in better socio-economic conditions, with a high level of education, health insurance, and living in neighbourhoods with basic sanitation, and knowing about HIV transmission forms, but with a risk behaviour to HIV infection. These findings are compatible with a study carried out among MSM in Mexico, Peru, and Brazil that showed that only 35% knew about autotest, and only 45.7% of them were tested for HIV in the last 6 months [20]. Another study among Brazilian MSMs also showed the prevalence of inconsistent condom usage with steady and casual partners at 64.5% and 50.8%, respectively [19].

Knowledge may not be converted into behavioural changes and can be influenced by several factors. For example, most of our sample were aware of PrEP, but the latest report showed that in 2023, there were only 965 PrEP users in all Pará [21]. In the history of HIV, homosexuality was labelled as a sin, a deviation from social norms, and dangerous [22]. A documentary study in Spain showed that the press changed the behaviour towards PrEP from a social improvement to a label of HIV-risk groups. This increased the stigma, decreased the search for a treatment, and decreased adherence to treatments [23].

Studies show that the LGBTQIA+ population still faces stigmatisation and discrimination and that health professionals are poorly qualified to meet their needs [24], violating the principles of the SUS and of the National LGBT Policy [10,11]. One way to combat such discrimination would be to report it via the “Dial 181” platform, which was unknown to most volunteers in this study. In addition, the dismantling of policies aimed at the LGBTQIA+ population between January 2019 and December 2021 during the Bolsonaro government negatively affected the guarantee of LGBT rights, contributing to an increase in homophobia in all social environments [25,26].

Other alarming findings were the low prevalence of HBV and HPV vaccinations among the participants and the large number of participants diagnosed with syphilis. Similar to our results, previous studies among 3178 MSMs from 12 Brazilian cities showed a HBV vaccination coverage rate of 74.4% [27] and a low national HPV vaccine coverage rate of 49.6% [28]. Furthermore, another study of 812 Brazilian MSMs who use dating applications showed a 34.23% prevalence of syphilis. Knowing that other STIs contribute to HIV transmission and disease progression [29,30], these are alarming results. And, considering this dangerous potential, in 2024, the Brazilian government expanded the age range for HPV vaccination from 9 to 45 years for those on PrEP or living with HIV [31].

Regarding the factors associated with behaviour and knowledge, the multivariate analysis showed that neither socioeconomic nor behavioural factors were associated with CP, but only FPs categorised on interpersonal and community levels. Not talking about their sex lives with confidants, not talking about their personal lives, not talking about STIs with work colleagues, and not taking part in NGOs contributed to lower scores concerning CP, suggesting the importance of the community belonging sense in the access to CP. A study among Canadian MSMs showed that social support was directly associated with a decrease in HIV risk behaviour and biomedical HIV prevention [32]. A study conducted among Brazilian MSMs found that those participating in NGOs were more aware of PEP and PrEP, were tested for HIV and syphilis more frequently, knew the healthcare centres providing rapid tests, and were more predisposed to obtaining counselling and knowledge about HIV [33]. According to the authors, in a world of extreme hostility towards the LGBT population, NGOs can promote a sense of belonging and establish a network of support and solidarity.

However, not being happy in their neighbourhood contributed to higher scores. Our hypothesis is that MSMs who are unhappy in their neighbourhood, perhaps because of homophobia, seek HIV prevention in TCCs for fearing the structural barriers in PHC serving their neighbourhood. A study among LGBT people in the city of Bahia, Brazil, showed that most of them experienced prejudice, stigma, and disrespect in PHC [13]. For being TCCs specialised in STI prevention, diagnosis, and treatment, it can lead MSMs to feel freer to seek knowledge and all preventive means against HIV without fearing discrimination. The territorialisation of PHCs has been a worrying factor in PrEP implementation because possible users could choose not to expose themselves to their acquaintances [34].

Due to the use of the TLS method for data collection, our sample consisted mainly of volunteers who had good living conditions, considering the large percentage of people living below the poverty line in the RMB. This may have influenced the non-association of socioeconomic factors with the dependent variable in our study. Therefore, future studies should consider stratifying the sample according to sociodemographic criteria. Additionally, to reduce the impact of social desirability in our data collection, besides explaining the importance of the study and the confidentiality of the data, we clarified that our interview was non-judgmental, and the interview was conducted in locations that ensured the volunteer’s privacy.

The fight against HIV in the Brazilian Amazon region faces challenges that differ from other Brazilian regions, such as the long distances that people must travel to access health services, often requiring boats, the rainy weather, and the low socioeconomic status of the population. Therefore, in addition to combating the HIV stigma, LGBTphobia, promoting health education for safe sexual behaviour, or publicizing all services offered by SUS towards STIs fighting, the more essential is to expand the number of healthcare centres offering PrEP, PEP, HIV treatment, and other STIs preventive and diagnostic services, as well as the relaxing territorialisation of the PHC.

## 5. Conclusions

Our results showed that although MSMs are aware of the ways in which HIV is transmitted, most volunteers display risky behaviours for HIV transmission, such as inconsistent use of condoms, not being tested every six months, not knowing about self-tests, and not being aware of the fact that condoms and lubricating gels are distributed by PHCs. Not talking about their sex lives with confidants and work colleagues, and not belonging to an NGO contributed to lower scores. On the other hand, not being happy in the neighbourhood where they lived contributed to higher scores.

## Figures and Tables

**Table 1:** Absolute (n) and relative frequency (%) of volunteers reaching maximum score for each factor comprising the dependent variable.

Domain	Variables	n	%
		(n = 384)	
Biomedical	Constant usage of condoms	82	21.4
	Usage of substances	88	22.90
	Knowledge about HIV transmission	378	98.44
	Knowledge about syphilis transmission	324	84.38
	Knowledge about autotest	181	47.25
	Knowledge about TCCs	159	41.39
	Knowledge about PHC offering rapid test	315	82.0
	Knowledge about PHC distributing condoms and lubricant	38	9.89
	At least one test for HIV every six months	145	37.76
	At least one test for syphilis every six months	45	11.72
	Never diagnosed with syphilis	193	50.26
	Diagnosed with syphilis and treated correctly	80	20.83
	Diagnosed with syphilis and not treated	111	28.91
	Aware of PEP	227	59.11
	Aware of PrEP	224	58.33
	Co-vaccinated against HBV and HPV	0	0
	Vaccinated against HBV	251	65.36
	Vaccinated only against HPV	93	24.22
Legal	“Dial 181”	112	29.16

**Table 2:** Socioeconomic factors and score of “behaviour/knowledge towards CP”.

Variables	n	%	Median	*p*
Age range				
18–29	97	24.48	8	0.587
30+	287	75.52	8	
City of residence				
Other	141	36.72	8	0.822
Belém	243	63.28	8	
Skin color				
Black/Blown	189	49.48	8	0.704
Yellow/White	195	50.52	8	
Marital status				
Single	215	55.91	8	0.258
Dating/Married	169	44.09	8	
Schooling level				
Elementary/High school	303	78.91	8	0.539
Graduated	81	21.09	8	
Monthly income (wages) *				
≤1	95	24.74	8	0.59
2–4	244	63.55	8	
5+	45	11.71	8	
Health insurance				
No	162	42.18	8	0.3
Yes	222	57.82	8	
Tattoos				
No	264	68.72	8	0.055
Yes	120	31.28	8	
Piercing				
No	290	70.34	8	0.372
Yes	94	29.66	8	
Victim of sexual abuse				
No	369	96.36	8	0.749
Yes	15	3.64	8	
Receive money for sex				
No	183	47.66	8	0.29
Yes	201	52.34	8	
Paid for sex				
No	316	80.29	8	0.634
Yes	68	19.71	8	
Have sex with someone living with HIV				
No	315	81.77	8	0.621
Yes	69	18.23	8	
Sex position				
Versatile	347	91.93	8	0.720
Bottom	22	5.73	8	
Top	15	2.34	7	
Use of dating applications				
No	47	12.24	8	0.875
Yes	337	87.76	8	

* 01 monthly Brazilian wage in 2024 = R$ 1412.00 (=U$232.90).

**Table 3:** Interpersonal protective factors and score of “behaviour/knowledge towards CP”.

Variables	n	%	Median	*p*
Religion				
Protestant	11	2.86	8	0.508
Catholic	108	28.12	8	
Other	92	23.97	8	
No religion	173	45.05	8	
Talk about sex life with confidants				
Never	86	22.40	8	0.084
Rarely/Sometimes/Almost Always	219	57.03	8	
Always	79	20.57	8	
Used to go to socialising places that talk about STIs */HIV ^#^ prevention				
No	173	45.08	8	0.549
Yes	211	54.92	8	
Know/talk to neighbours				
No	179	46.65	8	0.293
Yes	205	53.35	8	
Hang out with friends/colleagues living in the same neighbourhood				
No	184	47.90	8	0.777
Yes	200	52.01	8	
Job stability				
Unemployed	229	59.63	8	0.545
≤3 years	82	21.36	8	
>3years	73	19.01	8	
Talk about private life/STIs with work colleagues				
No	143	37.24	7	0.002
Yes	241	62.76	8	

* STIs—Sexually transmissible infections; ^#^ HIV—Human Immunodeficiency Virus.

**Table 4:** Community protective factors and score of “behaviour/knowledge towards CP”.

Variables	n	%	Median	*p*
Leisure options in the neighbourhood				
No	180	46.84	8	0.46
Yes	204	53.16	8	
Sports places in the neighbourhood				
No	171	44.52	8	0.463
Yes	213	55.48	8	
Neighbourhood with adequate rubbish collection				
No	172	44.74	8	0.31
Yes	212	55.26	8	
Neighbourhood with basic sanitation				
No	160	44.77	8	0.626
Yes	224	55.23	8	
Neighbourhood with adequate public transport				
No	171	44.52	8	0.463
Yes	213	55.48	8	
Participation in NGO *				
No	324	84.37	8	0.001
Yes	60	15.63	9	
Participates in the neighbourhood association				
No	191	49.74	8	0.234
Yes	193	50.26	8	
Being happy in the neighbourhood				
No	188	48.96	8	0.027
Yes	196	51.04	8	
Living in the same house >6 months.				
No	47	12.24	8	0.207
Yes	337	87.76	8	

* NGO—Non-governmental organisation.

**Table 5:** Policy protective factors and the score of “behaviour/knowledge towards CP”.

Variables	n	%	Median	*p*
Vote for politicians who support LGBT * causes				
No	173	45.04	8	0.11
Yes	211	54.96	8	
Demand action from the politicians you elect				
Neve	75	19.53	8	0.02
Rarely/Sometimes/Almost always	227	59.12	8	
Always	82	21.35	7	
Know the National LGBT Policy				
No	173	45.00	8	0.1
Yes	211	55.00	8	
Knows the principles that govern the SUS ^#^				
No	176	45.84	8	0.874
Yes	208	54.16	8	
Complain at the local when discriminated against				
No	177	46.04	8	0.017
Yes	207	53.96	8	

* LGBT—Lesbians, gays, bisexuals, and transgender people; ^#^ SUS—Unified Health Brazilian System.

**Table 6:** Protective factors associated with low and high scores of “behaviour/knowledge towards CP”.

Variables	Coefficient	95% CI	*p*	VIF
Constant	8.716	7.898; 9.534	<0.001	
Talk about sex life with confidant				
Never	−0.586	−1.15; −0.021	0.042	1.64
Talk about private life/^*^STIs with work colleagues				
No	−0.459	−0.845; −0.072	0.02	1.03
^#^NGO participation				
No	−0.758	−1. 292; −0.224	0.006	1.11
Being happy in the neighbourhood				
No	0.381	0.004; 0.758	0.048	1.03

CI—Confidence interval; VIF—Variance inflation factor; * STIs—Sexually transmissible infections; ^#^ NGO—Non-governmental organisation.

## Data Availability

Ethical restrictions may be applied to the public availability of the data. However, they can be obtained by contacting the corresponding author.

## Abbreviations

The following abbreviations are used in this manuscript:

PFs	Protective factors
CP	HIV combined prevention strategy
MSM	Men who have sex with men
PrEP	Pre-exposure prophylaxis
PEP	Post-exposure prophylaxis
ART	Antiretroviral treatment
HBV	Hepatitis B virus
HPV	Human papillomavirus
STIs	Sexually transmissible infections
PHC	Primary healthcare network
SUS	Unified Health Brazilian System

## Appendix A

**Table A1:** Socioeconomic, behavioural, and protective factors comprising the independent variables.

Category	Variables
Socioeconomic	Age range; City of residence; Skin colour/race; Marital status; Schooling level; Monthly income; Health insurance.
Behavioural	Tattoos; piercings; Victim of sexual abuse; Received money for sex; Paid for sex; Have sex with people living with HIV; Sex position; Use of dating applications.
Protective factors	
Interpersonal	Religion; Talk about your sex life with confidants; Socialising places that talk about STI/HIV prevention; Know/talk to neighbours; Go out with friends/colleagues living in the same neighbourhood; Job stability; Talk about private life/IST with work colleagues;
Communitary	Leisure options in the neighbourhood; Option to play sports in the neighbourhood; Neighbourhood with adequate rubbish collection; Neighbourhood with basic sanitation; Neighbourhood with adequate public transport. Participation in NGOs; Participates in the neighbourhood association; Reside in the same place longer than 6 months.
Public policy	Vote for politicians who support LGBT causes; Demand action from the politicians you elect; Know the National LGBT Policy; Know the principles that govern the SUS; Complain to the relevant bodies when discriminated against; Being happy in the neighbourhood.

**Table A2:** Correct answer for the questions composing the dependent variable “Knowledge/Behaviour toward CP” and their respective scores.

Questions	Score
Constant usage of condoms with casual and steady partners	1
No usage of substances associated with sex	1
Know that HIV can be transmitted by unprotected sex and from mother to child	1
Know that HIV can be transmitted by unprotected sex and from mother to child	1
Tested for HIV at least once every six months	1
Tested for HIV at least once every six months	1
Diagnosed and correctly treated for syphilis	1
Know the HIV autotest	1
Heard about PEP	1
Heard about PrEP	1
Know the TCC	1
Know the “Dial 181”	1
Know that rapid test are freely available at PHC	1
Know that lubricant and condoms are freely available at PHC	1
Co-vaccinated against HBV and HPV.	2
Vaccinated only against HBV	1
Vaccinated only against HPV	1
